# A combination of 7-ketocholesterol, lysosphingomyelin and bile acid-408 to diagnose Niemann-Pick disease type C using LC-MS/MS

**DOI:** 10.1371/journal.pone.0238624

**Published:** 2020-09-08

**Authors:** Chen Wu, Takeo Iwamoto, Mohammad Arif Hossain, Keiko Akiyama, Junko Igarashi, Takashi Miyajima, Yoshikatsu Eto

**Affiliations:** 1 Advanced Clinical Research Center, Institute of Neurological Disorders, Shin-Yurigaoka General Hospital, Kawasaki, Kanagawa, Japan; 2 Rare Disease Research Center, AnGes, Kawasaki, Kanagawa, Japan; 3 Core Research Facilities for Basic Science, The Jikei University School of Medicine, Tokyo, Japan; Fisheries and Oceans Canada, CANADA

## Abstract

**Background:**

Niemann-Pick disease type C (NPC) is an autosomal recessive disorder caused by mutations of *NPC1* or *NPC2*, which encode the proteins that are responsible for intracellular cholesterol trafficking. Loss of this function results in the accumulation of cholesterol-related products, such as oxysterols, sphingolipids, and NPC-related bile acids, which were recently used as biochemical biomarkers for the diagnosis of NPC. Bile acid-408 is a new significant compound we found in Japanese NPC patients, and it likely belongs to the category of bile acids. However, the diagnosis of NPC using a single biomarker is not satisfactory for clinical application because of the high instance of false negatives or positives. Therefore, we proposed an application of NPC diagnosis using a combination of 7-ketocholesterol (7-KC), lysosphingomyelin (lysoSM), bile acid-408 and/or glucosylsphingosine (lysoGL-1).

**Methods and findings:**

7-KC, lysoSM and lysoGL-1 in sera and bile acid-408 in dried blood spots (DBS) were quantified within 17 minutes using tandem mass spectrometry and high-resolution mass spectrometry, respectively. We measured these biomarkers in NPC patients (n = 19), X-linked adrenoleukodystrophy (X-ALD) patients (n = 5), patients with other lysosomal diseases (n = 300), newborns (n = 124) and healthy people (n = 74). Our results showed a promising accuracy (97%) for NPC diagnosis using the combination of 7-KC, lysoSM and bile acid-408. However, contrary to our expectations, lysoGL-1 levels did not present at a significantly greater amount in NPC patients than other patients and negative controls.

**Conclusions:**

The combination of 7-KC, lysoSM and bile acid-408 improves the accuracy of NPC diagnosis and is feasible for mass screening due to its simple sample preparation and measurement. Future research should investigate the chemical structure of bile acid-408 to further facilitate its advantage in diagnosis.

## 1. Introduction

Niemann-Pick disease type C (NPC, OMIM#607623, OMIM#601015) is a fatal, inherited lysosomal cholesterol storage disorder disease caused by the mutations of *NPC1* (95%) or *NPC2* (5%) [1–2]. The exact function of the proteins encoded by these genes is not certain, but NPC1 likely transfers low density lipoprotein-derived free cholesterol out of lysosomes or late endosomes, and NPC2 acts in concert with NPC1 [3–5]. Impaired intracellular lipid trafficking leads to an accumulation of unesterified cholesterol and sphingolipids, which were found in many tissues and organelles [1, 3, 6]. There is a huge heterogeneous clinical presentation of NPC, including its age of onset (from infants to adults) and its clinical phenotypes, which includes vertical supranuclear gaze palsy (VSGP), ataxia, dystonia, seizures, dysarthria, dysphagia and dementia [3, 7]. Although the age of onset varies significantly, the rate of progression appears linear independent of age of onset [3, 8, 9]. There are approximately 11 Japanese NPC newborns annually, with an incidence of 1.12:100,000 [10], and this number is likely to increase due to newborn screening of rare diseases in Japan. Miglustat is the only EMEA-approved drug for the treatment of NPC [11, 12], but substrate reduction therapy (SRT), chemical chaperones and gene therapy are under current investigation [13–16]. Recent clinical data on the efficacy of enzyme replacement therapy (ERT) on other lysosomal diseases, such as Gaucher disease and Fabry disease, indicated that disease prognoses improved dramatically when patients were treated early, especially before the onset of clinical phenotypes [17–20]. This treatment strategy may also be effective for NPC [5, 9]. Therefore, appropriate diagnostic methods are proposed.

A revised diagnostic algorithm for NPC was proposed with the initial screening use of an NPC suspicion index, genetic panel, and biomarker profiles instead of filipin staining followed by genetic analysis [21]. The primary drawbacks of previous processes are that they are not feasible for mass screening because they need some skills, and repeats of filipin staining are not always reliable [3, 6, 21]. Therefore, other methods to diagnose NPC, primarily the quantification of abnormal metabolites as biomarkers using mass spectrometry, has been widely used.

Oxysterols, lysosphingolipids and NPC-related bile acids in serum/plasma or dried blood spots (DBS) are biomarkers for the diagnosis of NPC [22–30]. Oxysterols are products of nonenzymatic cholesterol oxidation because of oxidative stress in NPC patients. Two significant oxysterols are used as plasma biomarkers, cholestane-3β,5α,6β-triol (C-triol) and 7-ketocholesterol (7-KC) [25, 27, 31, 32]. Lysosphingolipids are N-deacetylated sphingolipids, which are widely used in diagnosis of lysosomal disorders, such as globotriaosylsphingosine (lysoGb3) for Fabry disease [33] and glucosylsphingosine (lysoGL-1) for Gaucher disease [34]. For NPC, lysosphingomyelin (lysoSM), lysosphingomyelin-509 (lysoSM-509) and lysoGL-1 were reported for their significant performance in plasma or DBS [22, 26, 30, 35, 36]. A third category was also proposed as NPC-related bile acid-bile acid A/B and NPCBA1/BA2 [28, 29], likely from C-triol and 7-KC. We also found a significant new compound in Japanese NPC patients, bile acid-408. However, we have not confirmed its chemical structure at the date of this publication. The diagnosis of NPC using these compounds was reported in the US and Europe, but their applicability in Japanese NPC patients was not systemically presented. Furthermore, none of these biomarkers shows considerable advantages over the others. We proposed a biomarker-based diagnostic method using a combination of 7-KC, lysoSM and bile acid-408 to diagnose NPC.

## 2. Methods and materials

### 2.1 Samples

DBS (n = 56) and sera (n = 74) of anonymous controls (healthy adults) were obtained from Southern TOHOKU General Hospital, Japan. Anonymous newborn dried blood spots (NDBS; n = 124) and healthy adult DBS (n = 11) were provided by Osaka City University, Japan. Sera (n = 300) and DBS (n = 17) of patients with X-linked adrenoleukodystrophy (X-ALD) or other lysosomal diseases were collected by treating medical doctors across Japan. In addition, there were Japanese NPC patients with DBS (n = 16) and sera (n = 19) collected that were included in this cohort. All these samples were obtained with the participants’ full informed consent by their signature. The research was approved by ethical committee of Southern Tohoku Institute of Neuroscience (No.90-2, 256).

### 2.2 Preparation of samples

60 μL of serum was added into a 1.5 mL tube with 540 μL 2-Propanol (HPLC grade, Kanto, Japan) and a 20 μL mixture of deuterium-labeled internal standard (IS) 7-KC-d7, lysoSM-d7 and lysoGL-1-d5 (Avanti Polar Lipids, Inc., USA). After mixing for 2 min with a micro tube mixer (Tomy, Japan), the extraction was centrifugated for 20 min at 12,000g. Next, 480 μL of supernatant from this extraction was dried under an air stream and reconstituted with 20 μL of methanol (HPLC grade, Kanto, Japan). The final solution was transferred to autosampler micro vials for measurement. A mixture of IS was to normalize the process of sample preparation.

Three 3-mm punches of DBS/NDBS were put into a 1.5 mL tube containing 30 μL of MilliQ water. After mixing for 5 min, 690 μL of methanol and 30 μL IS mixture were added. Then the extraction was sonicated for 15 min at room temperature followed by centrifugation for 5 min at 10,000g. 600 μL of supernatant from this extraction was dried and reconstituted with 30 μL of methanol.

### 2.3 Calibration of 7-KC, lysoSM and lysoGL-1

Multicomponent calibration standards consisted of 7-KC, lysoGL-1 (Avanti Polar Lipids, Inc., USA) and lysoSM (Matreya LLC, USA) standards with the same amount of IS mixture in the methanol solvent. The calibrated amounts of 7-KC, lysoSM and lysoGL-1 were 0–0.8 ng (R^2^ = 0.997), 0–0.7 ng (R^2^ = 0.999) and 0–0.8 ng (R^2^ = 0.998), respectively. One calibration set was utilized for every 50 samples.

### 2.4 Quantification of 7-KC, lysoSM, lysoGL-1 by LC-MS/MS and bile acid-408 by LC-HRMS

7-KC, lysoSM and lysoGL-1 in serum were quantified by high-performance liquid chromatography (HPLC) connected to tandem mass spectrometry (LCMS-8040, Shimadzu, Japan). The mixture was eluted by mobile phase A (10 mM NH_4_COOH, Wako, Japan) and phase B (10% 10 mM NH_4_COOH/MeOH) through a C8 column (Inertsil C8–3, 3 μm, 2.1 × 50 mm, GL Sciences Inc., Japan) connected with an in-line filter unit (ACQUITY, Waters, USA). The multiple reaction monitoring (MRM) transitions were 401.3 > 95.1 m/z for 7-KC, 408.2 > 95.0 m/z for 7-KC-d7, 465.2 > 184.0 m/z for lysoSM, 472.2 > 184.0 for lysoSM-d7, 462.0 > 282.1 m/z for lysoGL-1 and 467.3 > 287.2 m/z for lysoGL-1-d5. The quantification of 7-KC, lysoGL-1 and lysoSM was performed by comparing the peak molecular target area with the IS area.

Bile acid-408 in DBS/NDBS was measured through high-performance liquid chromatography (HPLC) connected to high resolution mass spectrometry (HRMS, QTOF-Maxis3G, Bruker, Germany). The mixture was eluted by mobile phase A (H_2_O with 0.1% formic acid, Wako, Japan) and phase B (acetonitrile with 0.1% formic acid, Wako, Japan) through a C8 column connected with an in-line filter unit. The molecular ion of bile acid-408 is 409.1639 proposed as the form of [M+H]^+^. The amount of bile acid-408 was calculated based on IS 7-KC-d7.

Statistical analysis and plots were performed by GraphPad Prism 6. The scatter plot was graphed with error bar in the form of mean with standard deviation (SD). ROC-AUC curve was calculated on 95% confidence interval. The threshold for sensitivity and specificity was 49.80 ng/mL for 7-KC, 10.10 ng/mL for lysoSM, and 2.35 ng/punch for bile acid-408. Student’s t-test was performed by unpaired and two-tailed designs.

## 3. Results

### 3.1 7-KC

**Fig 1:**
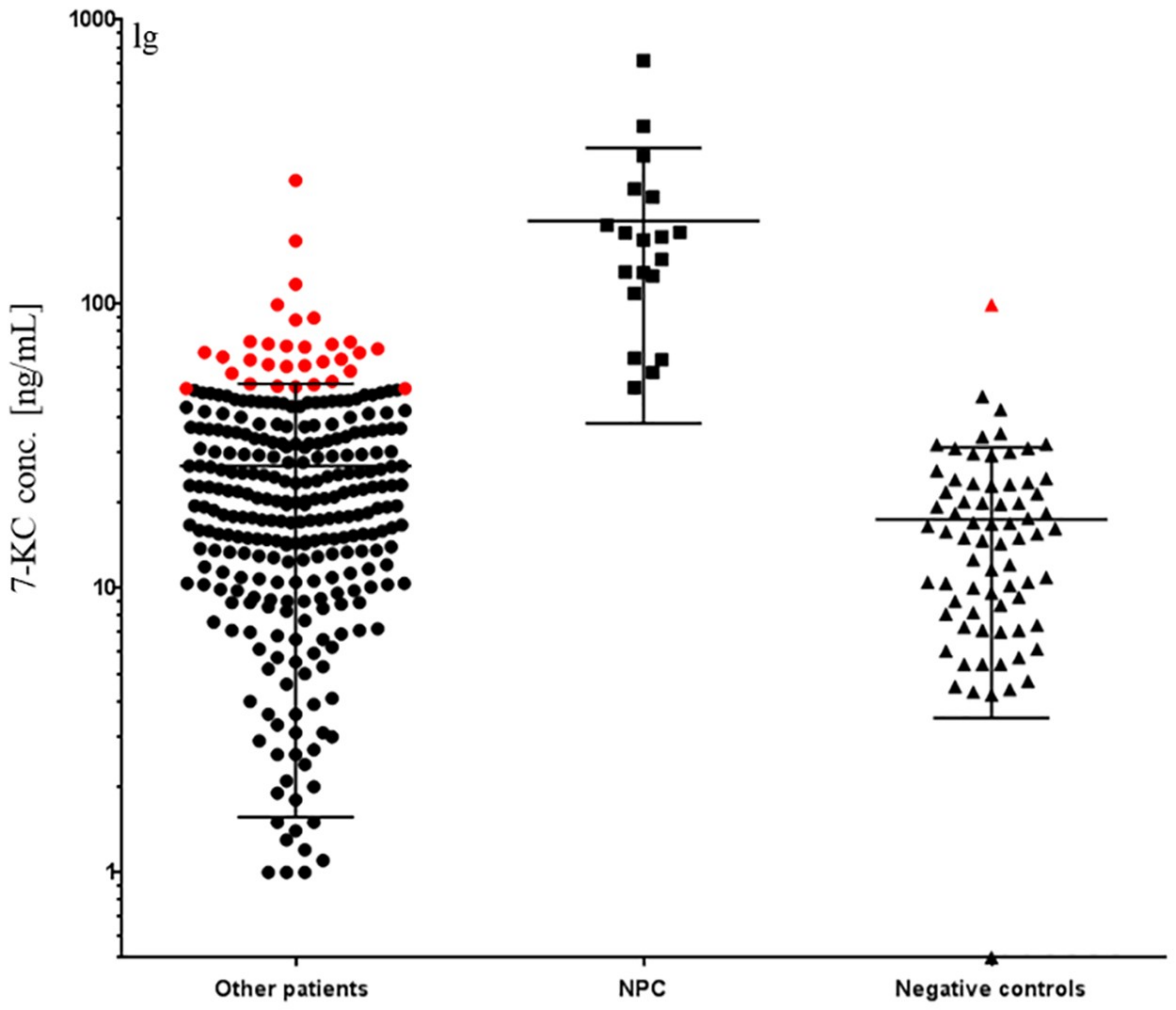
This is a plot of the amount of 7-KC in sera of NPC patients (n = 19), other patients (n = 295) and negative controls (n = 72). One point is one sample. The error bar is mean value with standard deviation (SD). Red: 7-KC amount above 50 ng/mL among other patients or negative controls.

7-KC levels in the sera of NPC patients, other patients (including X-ALD patients, Fabry patients, Gaucher patients and patients with other lysosomal diseases) and negative controls are shown in Fig 1 (sensitivity: 100%, specificity: 91%, ROC-AUC curve: 0.98, 95% CI: 0.97–1.00, t-test: 17.54, p<0.0001). The amount of 7-KC in NPC patients (n = 19) ranged from 51 ng/mL to 721 ng/mL, which are above the predetermined threshold of 50 ng/mL. However, the 7-KC levels of 32 samples from other patients (n = 295) and negative controls (n = 72) were also over this threshold, which resulted in an 8% false positive in the cohort. They included a Gaucher patient, a Mucopolysaccharidosis type IV (MPS 4) patient, 11 Fabry patients and 18 patients with other diseases. The two highest values above 100 ng/mL were Fabry patients with severe phenotypes. An abnormality in negative controls was a 32-year-old healthy male without clinical features prior to the sampling date.

### 3.2 LysoSM and lysoGL-1

**Fig 2:**
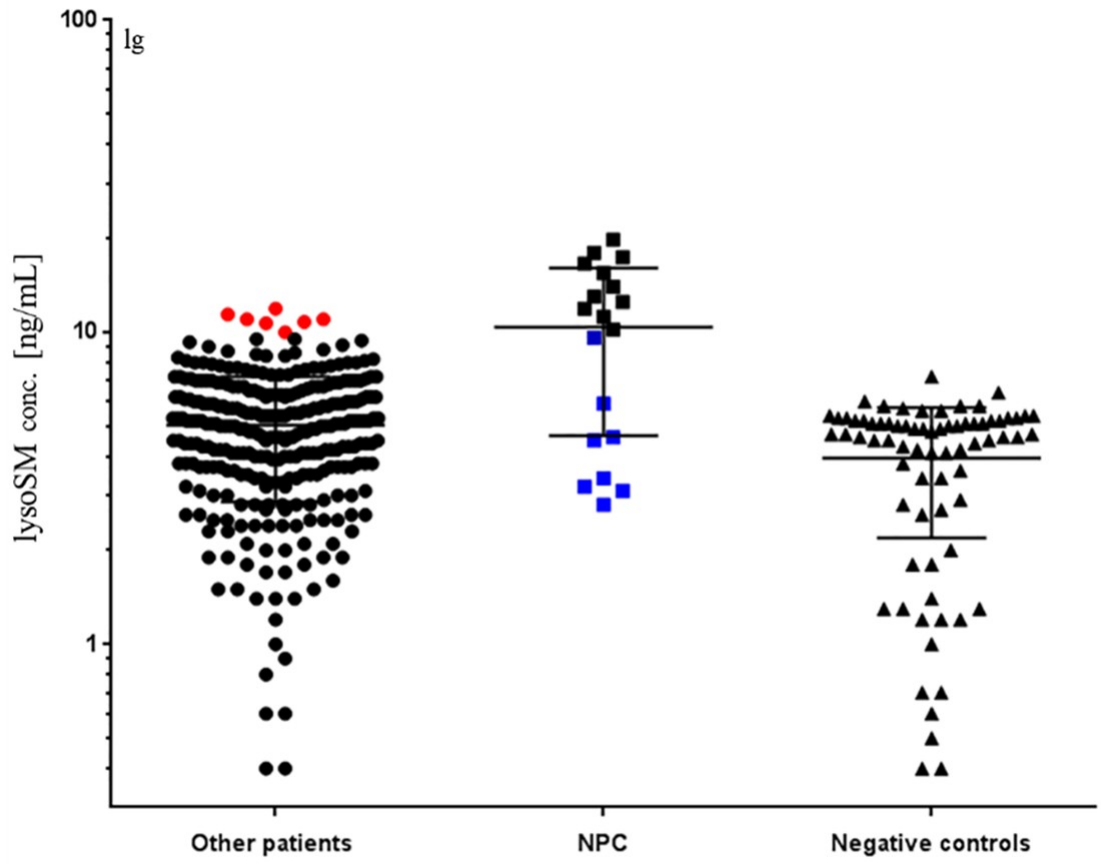
This is a plot of the amount of lysoSM in sera of NPC patients (n = 19), other patients (n = 300) and negative controls (n = 74). One point is one sample. The error bar is mean value with standard deviation (SD). Red: lysoSM amount over 10 ng/mL among other patients; blue: lysoSM amount below the threshold in NPC patients.

LysoSM levels in the sera of NPC patients (n = 19), other patients (n = 300) and negative controls (n = 74) were also measured (Fig 2). These levels were in the range of 2.8–19.8 ng/mL for NPC patients, 0.4–11.9 ng/mL for other patients, and 0.4–7.2 ng/mL for negative controls (sensitivity: 57.9%, specificity: 98.4%, ROC-AUC curve: 0.76, 95% CI: 0.61–0.91, t-test: 9.75, p<0.0001). Using the threshold of 10 ng/mL, there were 8 false negatives and 7 false positives. Among the 8 false negatives, there were 3 mild types with Miglustat treatment more than 2 years, 1 adult type, 3 without clinical features and 1 severe type with Miglustat treatment longer than 5 years. Regarding the 7 false positives, 5 were without clinical features, 2 were Fabry patients.

Another kind of lysosphingolipids is lysoGL-1, which is a common diagnostic biomarker for Gaucher disease, and its levels in sera were also determined. The results did not show a discrimination between NPC and other patients (S1 Fig), as presented by Welford R.W.D. et al. [22].

### 3.3 Bile acid-408

Bile acid-408 is a novel proposed compound that exhibited significance in the differentiation of Japanese NPC in our cohort. Initially, molecular ions at a range of 100 to 1000m/z from sera of 4 NPC patients and 48 healthy persons were investigated. According to the retention time, a molecular ion of 409.1639 m/z and MS/MS pattern suggested bile acid-408. However, its chemical structure has not been confirmed. Trihydroxy-cholestanoic acid (THCA, 3α,7α,12α-trihydroxycholestanoic acid, Avanti Polar Lipids, Inc., USA), a kind of bile acids, was not oxidized in the process of sample preparation, which was applied to bile acid-408.

**Fig 3:**
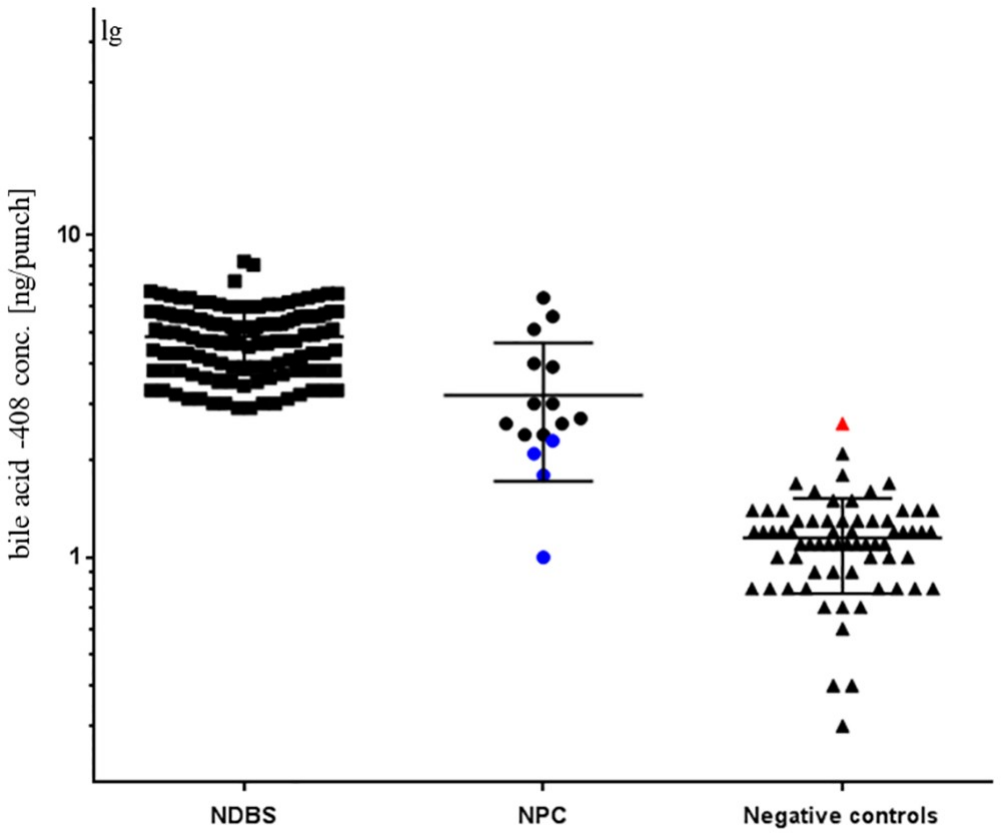
This is a plot of the amount of bile acid-408 in NDBS (n = 124), DBS of NPC patients (n = 16) and negative controls (n = 67). One point is one sample. The error bar is mean value with standard deviation (SD). Red: bile acid-408 amount over 2.4 ng/punch among negative controls; blue: bile acid-408 amount below the threshold in NPC patients.

The performance of bile acid-408 in NDBS (n = 124), DBS of NPC (n = 16) and negative controls (n = 67) was evaluated (Fig 3). These levels were generally much higher in NDBS than normal DBS samples. Comparison of NPC newborns and NDBS was not performed due to the lack of NPC newborn samples. Furthermore, NPC patients showed an increase in bile acid-408 levels, but with a 6% misdiagnosis rate based on lower 95% CI of the mean, 2.4 ng/punch (sensitivity: 75.0%, specificity: 98.5%, ROC-AUC: 0.95, 95% CI: 0.87–1.03, t-test: 10.25, p<0.0001). No clinical features were available on these 4 false negatives and 1 false positive.

### 3.4 Correlation

**Fig 4:**
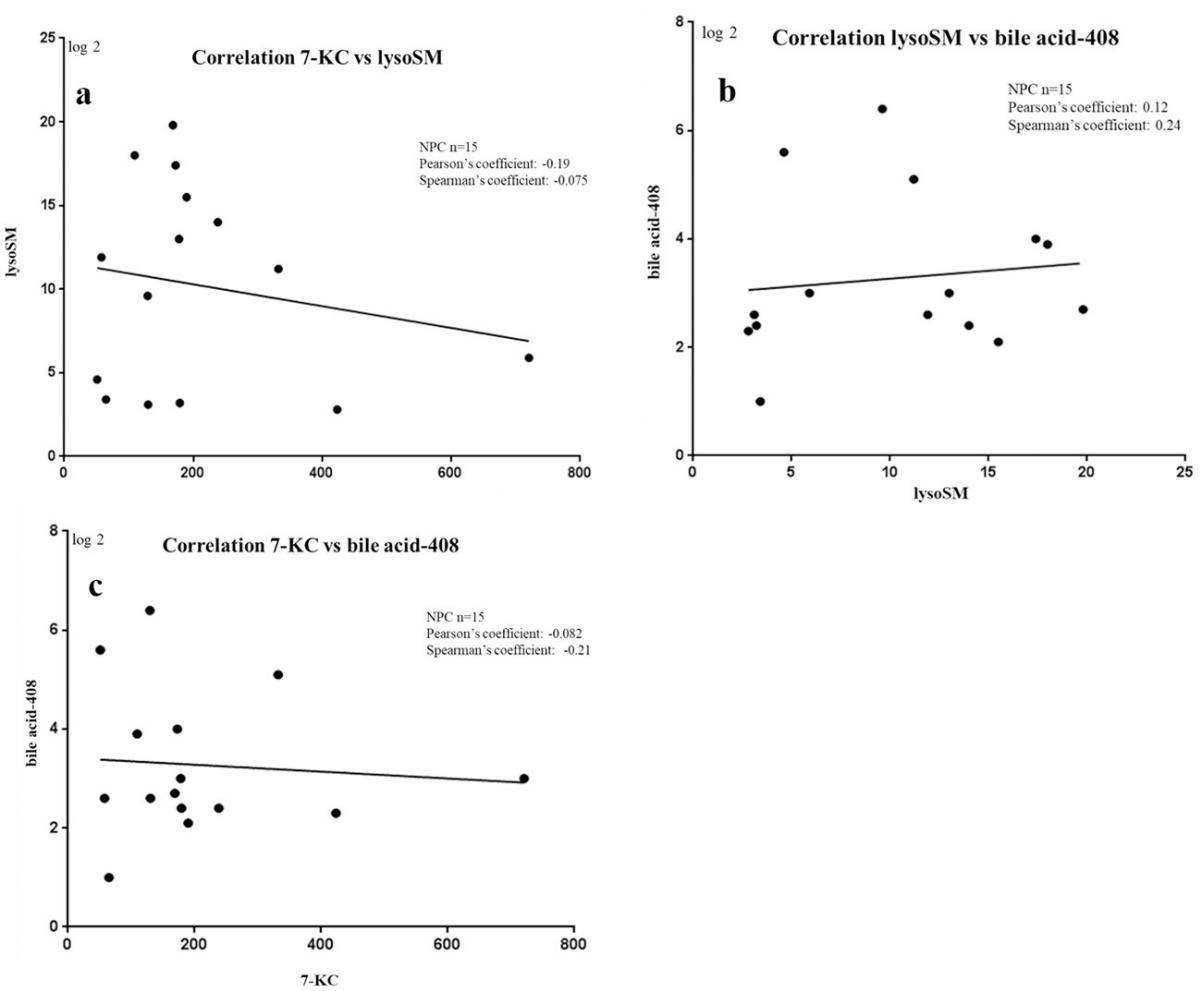
These are the plots of correlations between biomarkers. a: the correlation between 7-KC and lysoSM; b: the correlation between lysoSM and bile acid-408; c: the correlation between 7-KC and bile acid-408.

The correlations of these biomarkers were also investigated (Fig 4). According to Spearman’s and Pearson’s coefficients, there was no obvious correlation among these biomarkers.

### 3.5 Combinations

**Fig 5:**
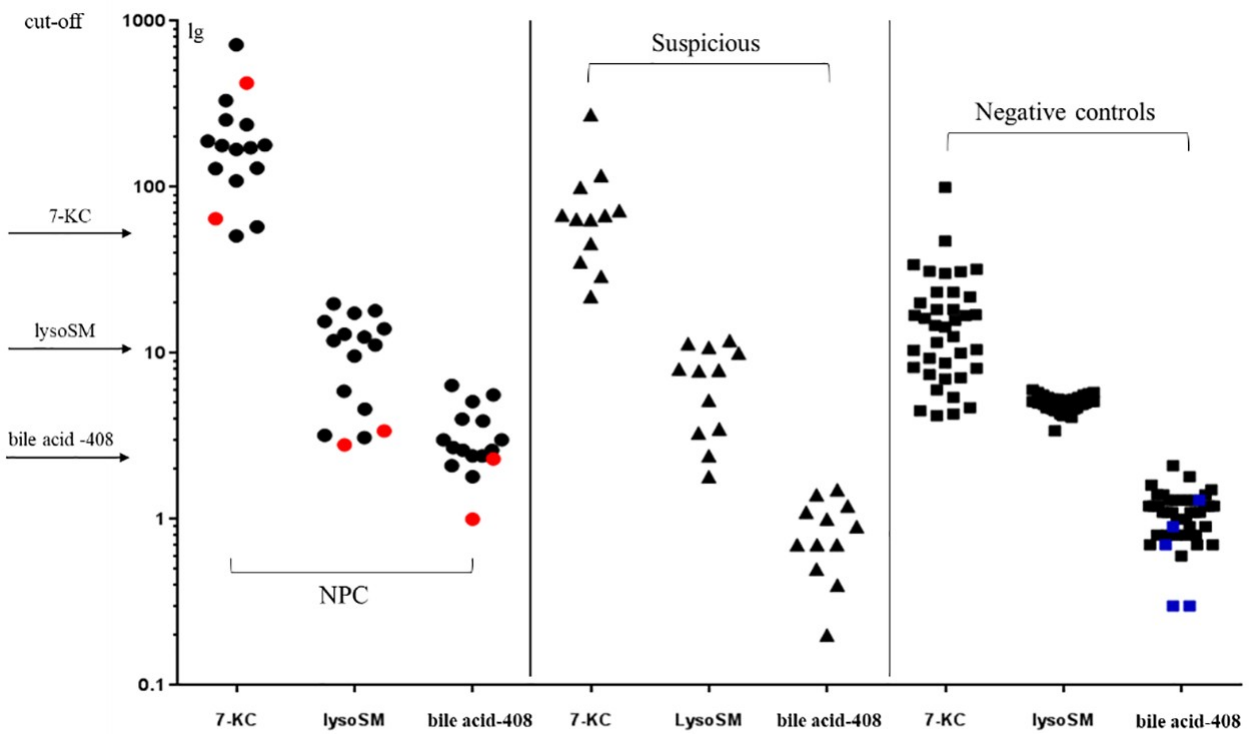
This is a plot of the combination of 7-KC, lysoSM and bile acid-408. One point is one sample. Left: NPC patients (n = 16); middle: high risk suspicious (n = 12); right: negative controls (n = 38) with X-ALD patients in blue (n = 5). Two NPC patients (red) had only one abnormal biomarker.

The efficiency of the combination of 7-KC, lysoSM and bile acid-408 in NPC diagnosis was evaluated in NPC patients (n = 16), high risk suspicious (n = 12) and negative controls (n = 38) with X-ALD patients (n = 5), and the results are presented in Fig 5. High risk suspicious (false positives) were selected that their 7-KC or lysoSM values were above the threshold, they did not have typical clinical features of NPC, and their DBS were also available. Abnormal accumulation of bile acids is related to peroxisomal diseases, therefore 5 X-ALD patients (a kind of peroxisomal diseases) were added to check the specificity of bile acid-408 to NPC. Threshold of each biomarker is also applied to the combinations. As the results, 14 of 16 NPC patients had at least two abnormal accumulated biomarkers, but all suspicious cases had only one biomarker. Two or more of three biomarkers in positives generated 100% sensitivity and specificity to NPC. However, 2 false negatives (2 NPC patients) had one abnormal biomarker resulting in 97% accuracy. In additional, X-ALD patients did not show significant amounts of bile acid-408 in their DBS.

## 4. Discussion

### 4.1 The combination of 7-KC, lysoSM and bile acid-408

Evaluation of 7-KC levels in sera/plasma of NPC patients was used for the diagnosis for several years, but its performance in DBS is not satisfactory. However, its stability is a main drawback as a biomarker. The amount of 7-KC in serum/plasma is easily elevated by high temperatures, which accelerate cholesterol (*ex vivo*) oxidization to 7-KC, such as the temperature fluctuation during transportation, sample preparation and cycles of freeze-thaw [24]. Some severe Fabry patients also exhibit high 7-KC levels in their sera, which magnified the false positives. The high levels were likely the result of the accumulation of endosomal-lysosomal cholesterol, which correlated with globotriaosylceramide (GB3).

LysoSM overcomes the problem of stability, but its application for DBS is not satisfactory. LysoSM-509, which probably belongs to the category of lysoSM, was reported for its sensitivity in DBS to diagnose NPC [36], but our results did not confirm its significance in DBS. Unlike 7-KC, lysoSM showed high false negatives, especially for patients who were treated with Miglustat for several years. Nevertheless, their clinical phenotypes were not improved obviously. LysoSM is easily degraded during prolonged storage time, and it is independent of age [23]. LysoSM levels were also high in the sera of severe Fabry patients, which may be indirectly caused by the storage of globotriaosylsphingosine (lysoGb3) in the metabolism of sphingolipids.

Bile acid-408 exhibits significant differentiation in DBS, but its chemical structure is under investigated in our laboratory. According to previous reports of bile acid A/ B and NPCBA1/BA2 [28, 29], bile acid-408 is likely to a third NPC-related bile acid. The results indicated that the threshold for newborn screening should be separate from juveniles and adults, that is, the amount of bile acid-408 in NDBS was greater than the lower 95% CI of NPC patients.

LysoGL-1 was previously thought to increase in NPC patient, but the content of lysoGL-1 in sera did not show any obvious difference between NPC patients and other patients in our study. This result did not confirm a previous publication [22].

The combination of three biomarkers provides a promising result that cannot be achieved with the use of any single biomarker. A new diagram of NPC diagnosis recently recommended the use of one biomarker for suspicious NPC followed by gene analysis and additional biomarker’ tests. It emphasized the importance of biomarkers, and our method has the advantages of being simple, effective, and applicable for mass or newborn screenings across Japan followed by gene analysis. All high risk suspicious in our cohort were screened out of NPC patients that they had only one abnormal biomarker, either 7-KC or lysoSM or bile acid-408 value above corresponding threshold. The diagnostic results were further confirmed by the gene analysis. Thus, we recommended that LysoSM and 7-KC in serum/plasma were measured simultaneously, and bile acid-408 in DBS was determined as a third confirmatory index. People with more than two biomarkers over their optimal thresholds are highly NPC patients.

### 4.2 Hypothesis of the metabolism of bile acid-408

Bile acids are primarily responsible for the absorption of fats and fat-soluble vitamins and the maintenance of cholesterol homeostasis [37]. Abnormalities in the metabolism of bile acids lead to several disorders, such as peroxisomal disease-Zellweger spectrum disorders (ZSDs) [37, 38]. Liver disease in ZSDs shows accumulation of dihydroxy-cholestanoic acid (DHCA) and trihydroxy-cholestanoic acid (THCA). X-ALD is also a peroxisomal disease, with the same diagnostic biomarker as ZSDs, 26:0 lysophosphatidylcholine (26:0 lysoPC) [39], but bile acid-408 in DBS of X-ALD patients did not present the significance we expected. There was no obvious correlation between 7-KC, lysoSM and bile acid-408 in our study. Therefore, bile acid-408 did not originate from 7-KC and lysoSM. Other reported NPC-related bile acids from 7-KC and C-triol were proposed [28, 29]. However, bile acid-408 is not likely to have the same metabolism pathway as other bile acids that are formed in peroxisomal diseases. The increase of bile acid-408 in NPC patients may be related to liver manifestations. One hypothesis is that bile acid-408 is involved in cholesterol transportation or degradation. Studies on the chemical structure and function of bile acid-408 should be performed in the future.

## 5. Conclusion

In summary, we propose a methodology using the combination of 7-KC, lysoSM and bile acid-408 for the diagnosis of NPC. The major advantages of the combination are its promising accuracy and its feasibility for mass screening. However, future investigations will confirm the chemical structure of bile acid-408.

## Supporting information

S1 Fig This is a plot of the amount of lysoGL-1 in sera of NPC patients (n = 4) and other patients (n = 207) including Gaucher patients (n = 3). One point is one sample. The error bar is mean value with standard deviation (SD). LysoGL-1 amount above 10 ng/mL are Gaucher patients.
(TIF)

S2 Fig These are chromatograms of 7-ketocholesterol, lysosphingomyelin, glucosylsphingosine and bile acid-408. a: chromatogram of 7-KC and 7-KC-d7 standards; b: chromatogram of lysoSM and lysoSM-d7 standards; c: a chromatogram of lysoGL-1 and lysoGL-d5 standards; d: a chromatogram of bile acid-408 and 7-KC-d7 in a DBS sample.
(TIF)

## Abbreviations

26:0 lysoPC: 26:0 lysophosphatidylcholine
7-KC: 7-ketocholesterol
C-triol: cholestane-3β,5α,6β-triol
DBS: dried blood spots
DHCA: dihydroxy-cholestanoic acid
ERT: enzyme replacement therapy
GB3: globotriaosylceramide
IS: internal standard
LC-HRMS: liquid chromatography connected to high resolution mass spectrometry
LC-MS/MS: liquid chromatography connected to tandem mass spectrometry
lysoGb3: globotriaosylsphingosine
lysoGL-1: glucosylsphingosine
lysoSM: lysosphingomyelin
MRM: multiple reaction monitoring
NDBS: newborn dried blood spots
NPC: Niemann-Pick disease type C
SRT: substrate reduction therapy
THCA: trihydroxy-cholestanoic acid
VSGP: supranuclear gaze palsy
X-ALD: X-linked adrenoleukodystrophy
ZSDs: Zellweger spectrum disorders

## References

[1] Vanier MT, Duthel S, Rodriguez-Lafrasse C, Pentchev P, Carstea ED. Genetic heterogeneity in Niemann-Pick C disease: a study using somatic cell hybridization and linkage analysis. Am J Hum Genet. 1996; 58: 118–125. 8554047 PMC1914948

[2] Patterson MC, Vanier MT, Suzuki K, Morris JA, Carstea E, Neufeld EB, et al. Niemann-Pick Disease Type C: A Lipid Trafficking Disorder. In: Valle D, Beaudet AL, Vogelstein B, Kinzler KW, Antonarakis SE, Ballabio A, Gibson KM, Mitchell G, editors. The Online Metabolic and Molecular Bases of Inherited Disease.

[3] Vanier MT. Niemann-Pick disease type C. Orphanet J Rare Dis. 2010; 5: 16 10.1186/1750-1172-5-16 20525256 PMC2902432

[4] Peak KB, Vance JE. Defective cholesterol trafficking in Niemann-Pick C-deficient cells. FEBS Lett. 2010; 584: 2731–2739. 10.1016/j.febslet.2010.04.047 20416299

[5] Zhang HW, Wang Y, Lin N, Yang R, Qiu WJ, Han LS, et al Diagnosis of Niemann-Pick disease type C with 7-ketocholesterol screening followed by NPC1/NPC2 gene mutation confirmation in Chinese patients. Orphanet J Rare Dis. 2014; 9: 82 10.1186/1750-1172-9-82 24915861 PMC4059728

[6] Vanier MT, Gissen P, Bauer P, Coll MJ, Burlina A, Hendriksz CJ, et al Diagnostic tests for Niemann-Pick disease type C (NP-C): A critical review. Mol Genet Metab. 2016; 118: 244–254. 10.1016/j.ymgme.2016.06.004 27339554

[7] Patterson M. Niemann-Pick Disease Type C Synonym: Juvenile Niemann-Pick Disease. In: Pagon RA, Adam MP, Ardinger HH, et al., editors. GeneReviews^®^ [Internet]. Seattle (WA): University of Washington, Seattle; 1993–2019. 20301473

[8] Yanjanin NM, Vélez JI, Gropman A, King K, Bianconi SE, Conley SK, et al Linear Clinical Progression, Independent of Age of Onset, in Niemann-Pick Disease, Type C. Am J Med Genet B Neuropsychiatr Genet. 2010; 153B (1): 132–140. 10.1002/ajmg.b.30969 19415691 PMC2798912

[9] Stampfer M, Theiss S, Amraoui Y, Jiang XT, Keller S, Ory DS, et al Niemann-Pick disease type C clinical database: cognitive and coordination deficits are early disease indicators. Orphanet J Rare Dis. 2013; 8: 35 10.1186/1750-1172-8-35 23433426 PMC3649939

[10] Wassif CA, Cross JL, Iben J, Sanchez-Pulido L, Cougnoux A, Platt FM, et al High incidence of unrecognized visceral/neurological late-onset Niemann-Pick disease, type C1, predicted by analysis of massively parallel sequencing data sets. Genet. Med. 2016; 18: 41–48. 10.1038/gim.2015.25 25764212 PMC4486368

[11] Patterson MC, Vecchio D, Prady H, Abel L, Wraith JE. Miglustat for treatment of Niemann-Pick C disease: a randomised controlled study. Lancet Neurol. 2007; 6 (9): 765–72. 10.1016/S1474-4422(07)70194-1 17689147

[12] Patterson MC, Hendriksz CJ, Walterfang M, Sedel F, Vanier MT, Wijburg F, et al Recommendations for the diagnosis and management of Niemann-Pick disease type C: an update. Mol Genet Metab. 2012; 106 (3): 330–44. 10.1016/j.ymgme.2012.03.012 22572546

[13] Rosenbaum AI, Maxfield FR. Niemann-Pick type C disease: molecular mechanisms and potential therapeutic approaches. J Neurochem. 2011; 116 (5): 789–795. 10.1111/j.1471-4159.2010.06976.x 20807315 PMC3008286

[14] Bremova T, Malinová V, Amraoui Y, Mengel E, Reinke J, Kolníková M, et al Acetyl-dl-leucine in Niemann-Pick type C: A case series. Neurology. 2015; 85 (16): 1368–1375. 10.1212/WNL.0000000000002041 26400580

[15] Cougnoux A, Cluzeau C, Mitra S, Li R, Willimans I, Burkert K, et al Necroptosis in Niemann-Pick disease, type C1: a potential therapeutic target. Cell Death Dis. 2016; 7: e2147 10.1038/cddis.2016.16 26986514 PMC4823930

[16] Chandler RJ, Williams IM, Gibson AL, Davidson CD, Incao AA, Hubbard BT, et al Systemic AAV9 gene therapy improves the lifespan of mice with Niemann-Pick disease, type C1. Hum Mol Genet. 2017; 26 (1): 52–64. 10.1093/hmg/ddw367 27798114 PMC6075521

[17] Stirnemann J, Belmatoug N, Camou F, Serratric C, Froissart R, Caillaud C, et al A Review of Gaucher Disease Pathophysiology, Clinical Presentation and Treatments. Int. J. Mol. Sci. 2017; 18 (2): pii: E441 10.3390/ijms18020441 28218669 PMC5343975

[18] Pastores GM and Hughes DA. Gaucher Disease Synonyms: Glucocerebrosidase Deficiency, Glucosylceramidase Deficiency. In: Pagon RA, Adam MP, Ardinger HH, et al., editors. GeneReviews^®^ [Internet]. Seattle (WA): University of Washington, Seattle; 1993–2019. 20301446

[19] Hopkin RJ, Jefferies JL, Laney DA, Lawson VH, Mauer M, Taylor MR, et al The management and treatment of children with Fabry disease: A United States-based perspective. Mol Genet Metab. 2016; 117 (2): 104–113. 10.1016/j.ymgme.2015.10.007 26546059

[20] Ortiz A, Germain DP, Desnick RJ, Politei J, Mauer M, Burlina A, et al Fabry disease revisited: Management and treatment recommendations for adult patients. Mol Genet Metab. 2018; 123 (4): 416–427. 10.1016/j.ymgme.2018.02.014 29530533

[21] Patterson M, Clayton P, Gissen P, Anheim M, Bauer P, Bonnot O, et al Recommendations for the detection and diagnosis of Niemann-Pick disease type C: An update. Neurol Clin Pract. 2017; 7 (6): 499–511. 10.1212/CPJ.0000000000000399 29431164 PMC5800709

[22] Welford RWD, Garzotti M, Lourenço CM, Mengel E, Marquardt T, Reunert J, et al Plasma Lysophingomyelin Demonstrates Great Potential as a Diagnostic Biomarker for Niemann-Pick Disease Type C in a Restrospective Study. PLoS ONE. 2014; 9 (12): e114669 10.1371/journal.pone.0114669 25479233 PMC4257710

[23] Chuang WL, Pacheco J, Cooper S, McGovern MM, Cox GF, Keutzer J, et al Lyso-sphingomyelin is elevated in dried blood spots of Niemann-Pick B patients. Mol Genet Metab. 2014; 111: 209–211. 10.1016/j.ymgme.2013.11.012 24418695

[24] Boenzi S, Deodato F, Taurisano R, Martinelli D, Verrigni D, Carrozzo R, et al A new simple and rapid LC-ESI-MS/MS method for quantification of plasma oxysterols as dimethylaminobutyrate esters. Its successful us for the diagnosis of Niemann-Pick type C disease. Clin Chim Acta. 2014; 437: 93–100. 10.1016/j.cca.2014.07.010 25038260

[25] Reunert J, Lotz-Havla AS, Polo G, Kannernberg F, Fobker M, Griese M, et al Niemann-Pick Type C-2 Disease: Identification by Analysis of Plasma Cholestane-3ß,5α,6ß-Triol and Further Insight into the Clinical Phenotype. JIMD Rep. 2015; 23: 17–26. 10.1007/8904_2015_423 25772320 PMC4484906

[26] Giese AK, Mascher H, Grittner U, Eichler S, Kramp G, Lukas J, et al A novel, highly sensitive and specific biomarker for Niemann-Pick type C1 disease. Orphanet J Rare Dis. 2015; 10: 78 10.1186/s13023-015-0274-1 26082315 PMC4479076

[27] Reuner J, Fobker M, Kannenberg F, Chesne ID, Plate M, Wellhausen J, et al Rapid Diagnosis of 83 Patients with Niemann Pick Type C Disease and Related Cholesterol Transport Disorders by Cholestantriol Screening. EBioMedicine. 2016; 4: 170–175. 10.1016/j.ebiom.2015.12.018 26981555 PMC4776073

[28] Jiang XT, Sidhu R, Mydock-McGrane L, Hsu FF, Covey DF, Scherrer DE, et al Development of a bile acid-based newborn screen for Niemann-Pick disease type C. Sci Transl Med. 2016; 8 (337): 337ra63 10.1126/scitranslmed.aaf2326 27147587 PMC5316294

[29] Mazzacuva F, Mills P, Mills K, Camuzeaux S, Gissen P, Nicoli ER, et al Identification of novel bile acids as biomarkers for the early diagnosis of Niemann-Pick C disease. FEBS Lett. 2016; 590: 1651–1662. 10.1002/1873-3468.12196 27139891 PMC5089630

[30] Pettazzoni M, Froissart R, Pagan C, Vanier MT, Ruet S, Latour P, et al LC-MS/MS multiplex analysis of lysosphingolipids in plasma and amniotic fluid: A novel tool for the screening of sphingolipidoses and Niemann-Pick type C disease. PLoS ONE. 2017; 12 (7): e0181700 10.1371/journal.pone.0181700 28749998 PMC5531455

[31] Jiang XT, Sidhu R, Porter FD, Yanjanin NM, Speak AO, Taylor te Vruchte D, et al A sensitive and specific LC-MS/MS method for rapid diagnosis of Niemann-Pick C1 disease from human plasma. J. Lipid Res. 2011; 52: 1435–1445. 10.1194/jlr.D015735 21518695 PMC3122908

[32] Boenzi S, Deodato F, Taurisano R, Goffredo BM, Rizzo C, Dionisi-Vici C, et al Evaluation of plasma cholestane-3β,5α,6β-triol and 7-ketocholesterol in inherited disorders related to cholesterol metabolism. J. Lipid Res. 2016; 57: 361–367. 10.1194/jlr.M061978 26733147 PMC4766985

[33] Nowak A, Mechtler TP, Desnick RJ, Kasper DC. Plasma LysoGb3: A useful biomarker for the diagnosis and treatment of Fabry disease heterozygotes. Mol Genet Metab. 2017; 120: 57–61. 10.1016/j.ymgme.2016.10.006 27773586

[34] Rolfs A, Giese AK, Grittner U, Mascher D, Elstein D, Zimran A, et al Glucosylsphingosine Is a Highly Sensitive and Specific Biomarker for Primary Diagnostic and Follow-Up Monitoring in Gaucher Disease in a Non-Jewish, Caucasian Cohort of Gaucher Disease Patients. PLoS ONE. 2013; 8 (11): e79732 10.1371/journal.pone.0079732 24278166 PMC3835853

[35] Mashima R, Maekawa M, Narita A, Okuyama T, Mano N. Elevation of plasma lysosphingomyelin-509 and urinary bile acid metabolite in Niemann-Pick disease type C-affected individuals. Mol Genet Metab Rep. 2018; 15: 90–95. 10.1016/j.ymgmr.2018.03.005 30023294 PMC6047109

[36] Kuchar L, Sikora J, Gulinello ME, Poupetova H, Lugowska A, Malinova V, et al Quantitation of plasmatic lysosphingomyelin and lysosphingomyelin-509 for differential screening of Niemann-Pick A/B and C disease. Anal Biochem. 2017; 525: 73–77. 10.1016/j.ab.2017.02.019 28259515

[37] Vaz FM, Ferdinandusse S. Bile acid analysis in human disorders of bile acid biosynthesis. Mol Aspects Med. 2017; 56: 10–24. 10.1016/j.mam.2017.03.003 28322867

[38] Klouwer FCC, Berendse K, Ferdinandusse S, Wanders RJA, Engelen M, Poll-The BT. Zellweger spectrum disorders: clinical overview and management approach. 10.1186/s13023-015-0368-9 PMC4666198 26627182

[39] Klouwer FCC, Ferdinandusse S, Lenthe HV, Kulik W, Wanders RJA, Poll-The BT, et al Evaluation of C26:0-lysophosphatidylcholine and C26:0-carnitine as diagnostic markers for Zellweger spectrum disorders. J Inherit Metab Dis. 2017; 40:875–881. 10.1007/s10545-017-0064-0 28677031

