# Supplementary figures and images for: A combination of 7-ketocholesterol, lysosphingomyelin and bile acid-408 to diagnose Niemann-Pick disease type C using LC-MS/MS
Source: PLoS One. 2020 Sep 8;15(9):e0238624. doi: 10.1371/journal.pone.0238624 (PMC7478639; doi:10.1371/journal.pone.0238624)

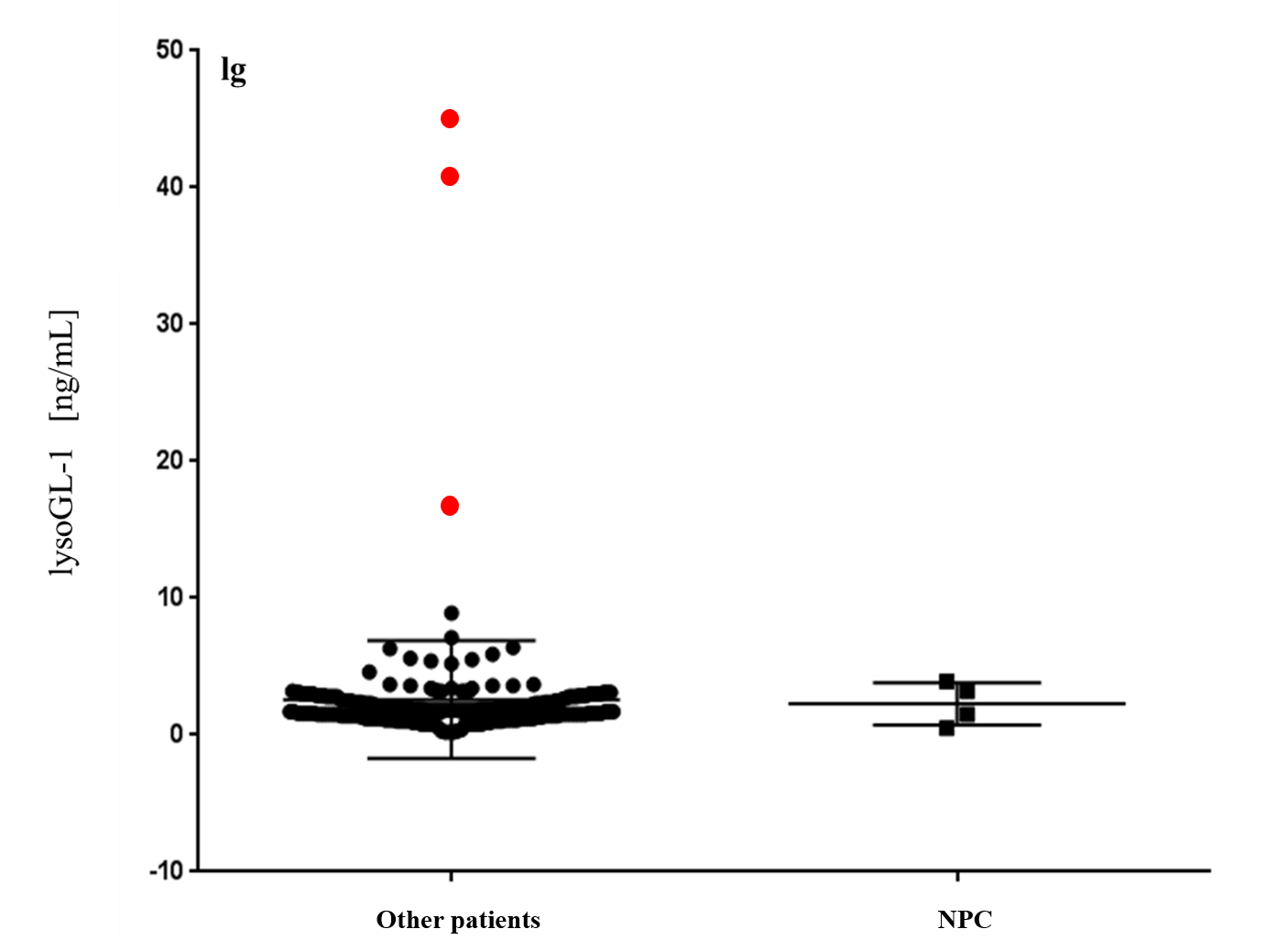

Supplement: S1 Fig — One point is one sample. The error bar is mean value with standard deviation (SD). LysoGL-1 amount above 10 ng/mL are Gaucher patients. (TIF) [file pone.0238624.s001.tif]

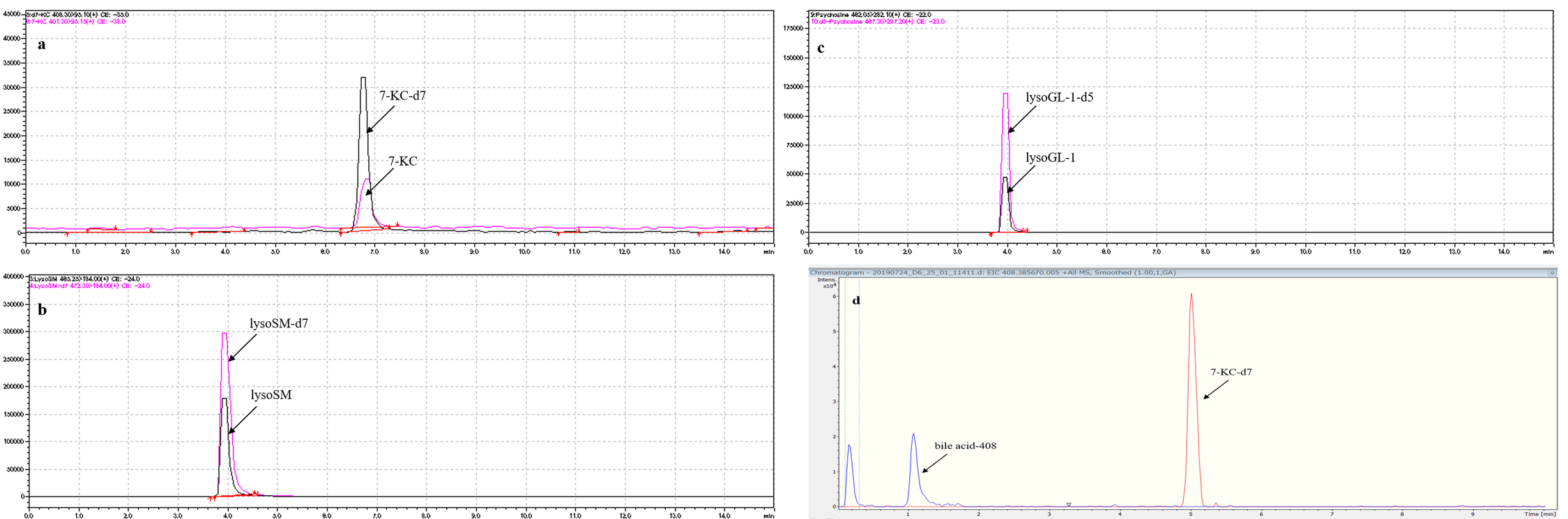

Supplement: S2 Fig — a: chromatogram of 7-KC and 7-KC-d7 standards; b: chromatogram of lysoSM and lysoSM-d7 standards; c: a chromatogram of lysoGL-1 and lysoGL-d5 standards; d: a chromatogram of bile acid-408 and 7-KC-d7 in a DBS sample. (TIF) [file pone.0238624.s002.tif]
